# Pilot randomised controlled trial of protective socks against usual care to reduce skin tears in high risk people: ‘stopcuts’

**DOI:** 10.1186/1745-6215-16-S2-P20

**Published:** 2015-11-16

**Authors:** Roy Powell, Christopher Hayward, Caroline Snelgrove, Kathleen Polverino, Linda Park, Rohan Chauhan, Philip Evans, Rachel Byford, Carolyn Charman, Christopher Foy, Andrew Kingsley

**Affiliations:** 1 Research and Development Directorate, Royal Devon and Exeter NHS Foundation Trust, Exeter, UK; 2 Peninsula Clinical Trials Unit (PenCTU), Plymouth, UK; 3 University of Exeter Medical School, Exeter, UK; 4 NIHR Clinical Research Network, South West Peninsula, Exeter, UK; 5 Research and Development Office, Gloucester Royal Hospital, Gloucester, UK; 6 Northern, Eastern and Western Devon Clinical Commissioning Group, Exeter, UK

## 

Skin tears are traumatic injuries occurring mostly on the extremities due to shearing and friction forces that separate the epidermis and the dermis from underlying tissues. They are common in older adults - especially those who have taken long-term steroids - and are caused by falls, mobility aids and knocks from obstacles. Traumatic injuries are the second most common type of wounds in care homes and are the commonest reason for community nurse involvement. Pretibial skin tears can develop into leg ulcers, which require lengthy, expensive treatment. No effective prevention exists. We are trialling knee-length, protective socks that contain cut, tear and abrasion-resistant Kevlar fibres and cushioning layers.

In this pilot parallel group, randomised controlled trial, 90 people at risk of skin-tear injury were recruited in Devon from care homes and the community and were randomised to wear the intervention socks or usual clothing for 4 months. The aim of the pilot was to inform the design of a definitive trial (that recruitment, randomisation, treatment and follow-up ran smoothly).

395 patients were approached and 90 were consented (54 in care homes and 36 community). Median age of participants was 85 years. 31 skin tear injuries occurred in 18 (20%) of the 90 participants over a period of 112 days. There were 21 injuries among 10 (21.74%) patients in the control group (n=46) and 10 injuries among 8 (18.18%) patients in the socks group (n=44). Only two in this group were definitely wearing their intervention socks at the time of their injury.

